# GBD2021: headache disorders and global lost health – a focus on children, and a view forward

**DOI:** 10.1186/s10194-024-01795-2

**Published:** 2024-06-03

**Authors:** Timothy J Steiner, Andreas Husøy, Lars Jacob Stovner

**Affiliations:** 1 Department of Neuromedicine and Movement Science, Norwegian University of Science and Technology, Edvard Griegs gate, Trondheim, Norway; 2 Department of Neurology, University of Copenhagen, Copenhagen, Denmark; 3 Division of Brain Sciences, Imperial College London, London, UK

**Keywords:** Global Burden of Disease study, Headache disorders, Migraine, Tension-type headache, Children, Epidemiology, Burden of headache, Population health, Global Campaign against Headache

The Global Burden of Disease (GBD) study 2019, the last iteration prior to now, found migraine to be the second highest cause, globally, of years lived with disability (YLDs) [1, 2], a measure of lost health attributed to non-fatal disease [3, 4]. In young women (aged < 50 years), it was the first cause [1, 2]. These were not new findings, since previous GBD iterations had found very much the same. In GBD2016, neurological disorders accounted for 8.6% of all global YLDs, headache disorders for more than three quarters of all neurological YLDs (6.5% of all YLDs), and migraine for 86% of these (5.6% of all YLDs) [5, 6]. The public-health message has remained the same throughout: migraine is among the very top causes of health loss in the productive populations of the world. So has the political message that much of this health loss is remediable, and should be a high priority for remedy, since treatments are widely available and highly cost-effective [7].

Self-evidently, these messages have remained unheard, but it is not our purpose here to repeat them.

Of course, health politicians recognise YLDs as only a partial measure of lost health, taking no account of mortality – to which headache disorders do not contribute. It is not difficult to understand that disease-attributed early mortality attracts attention. GBD2019 focused on disability-adjusted life years (DALYs), the sum of YLDs and years of life lost (YLLs) to early mortality. Headache disorders ranked 14th among global causes of DALYs (both genders, all ages), 10th among females, third among young adults (15–49 years) (to low back pain and ischaemic heart disease), and second among young adult females (to low back pain) [1].

Does GBD2021, just published [8], add anything new regarding headache? The key findings are in Table 1, taken from the on-line data source [9]. At level 3 (groups of related disorders), headache disorders are now ranked 15th cause of DALYs (608/100,000 of the population; 1.65% of all DALYs). Among young adults they are fourth (3.63% of all DALYs among this age group) – displaced by COVID-19, the top cause, and depressive disorders. Among children (5–14 years) they are ninth (3.09% of all DALYs). At level 4 (individual disorders), migraine is just within the top 20 causes of DALYs (1.49%), but fourth among young adults and third among young females.

Table 1 shows rates are the same for DALYs and YLDs – no mortality is recorded for headache disorders. At level 3, headache disorders remain the third cause of YLDs (5.23%), behind low back pain – which is some way ahead (7.75%) – and depressive disorders (6.22%), these constantly being the top three. Among young adults, headache disorders remain second (7.65%; depressive disorders 8.41%), but among young females (8.16%) they are displaced to third by gynaecological disorders (9.26%). At level 4, migraine is now fourth among all ages (4.73%), pushed down by major depression (5.09%, with a 13.5% increase since GBD2019) and age-related hearing loss (4.89%, up 1.9%). Among young adults, migraine (6.98%) is second to low back pain (7.67%), but overtaken also by major depression among young males (6.39% *versus* 6.27%).

**Table 1:** Key findings of GBD2021 in relation to headache disorders (from [9])

	Disability-adjusted life years (DALYs)			Years lived with disability (YLDs)		
	Rank^1^	Rate per 100,000	Proportion of all (%)	Rank^1^	Rate per 100,000	Proportion of all (%)
**Headache disorders** ^**2**^						
Both genders, all ages	15th	608	1.65	3rd	608	5.23
males	24th	468	1.19	5th	468	4.58
females	10th	749	2.20	3rd	749	5.75
Young adults (15–49 years)	4th	824	3.63	2nd	824	7.65
males	12th	636	2.65	2nd	636	6.98
females	4th	1,017	4.78	3rd	1,017	8.16
Children (5–14 years)	9th	277	3.09	4th	277	5.55
males	14th	215	2.37	7th	215	4.56
females	4th	343	3.89	3rd	343	6.48
**Migraine** ^**3**^						
Both genders, all ages	19th	550	1.49	4th	550	4.73
males	22nd	417	1.06	6th	417	4.08
females	14th	684	2.00	3rd	684	5.24
Young adults (15–49 years)	4th	752	3.32	2nd	752	6.98
males	9th	572	2.38	3rd	572	6.27
females	3rd	936	4.40	2nd	936	7.51
Children (5–14 years)	8th	257	2.87	4th	257	5.15
males	13th	197	2.17	7th	197	4.18
females	4th	322	3.65	3rd	322	6.08

^1^Rank according to rate per 100,000; ^2^at level 3 of GBD (groups of related disorders); ^3^at level 4 of GBD (individual disorders)

There has been little focus, in past iterations of GBD, on children (here considered as those aged 5–14 years). The data for this age group are not good, largely because of a paucity of reliable studies. This is now being remedied, with a series of schools-based studies [10, 11] – probably the most reliable means of gathering population-based data in this age group [12]. Meanwhile, although incomplete, the information offered on children by GBD2021 [9, 13] is thought-provoking (Table 1). Headache disorders are among the top ten causes (3.09%) of *DALYs* in 5–14-year-olds, fourth (3.89%) among females (behind dietary iron deficiency [7.41%], anxiety disorders [4.98%] and diarrhoeal diseases [4.19%]). They are fourth highest cause of YLDs (5.55%), behind dietary iron deficiency (10.50%), neonatal disorders (7.28%) and anxiety disorders (7.16%).

Headache disorders among children raise especial concerns for three reasons. While the first is that they represent a cause of current ill health, the others are future-directed. Headache disorders in childhood are harbingers of headache in adulthood, which, perhaps, intervention in childhood can mitigate. And headache disorders in childhood interfere with education, potentially with long-term deleterious consequences [14]. Headache disorders in children and adolescents demand much more attention.

Table 2 banishes any remaining perception that migraine is a disorder of importance only in high-income countries. The ranking is variable for DALYs, and lowest in low-income countries, with the proportion of all DALYs that are attributable to migraine showing a clear gradient downwards from high-income to low-income. A similar gradient – not quite so clear – is seen in rates per 100,000 of the population, and reflects but is not wholly explained by prevalence estimates: 10.5% in low-income, 15.2% in lower-middle, 14.2% in upper-middle and 16.3% in high-income countries. The quantity and quality of data from low- and lower-middle-income countries, although recently much enhanced [10], remain much poorer than in high-income countries, and estimates for the first two categories may still be affected by incomplete case ascertainment. But YLDs tell a slightly different story: although the same gradient is seen in rates per 100,000, migraine ranks fourth or fifth in all but high-income countries, where it ranks eighth (Table 2). Proportionately, migraine is least in high-income countries, and greatest in lower-middle. There are complex conflicting influences that may be difficult to unravel, many if not most arising from other diseases, but the message from Table 2 is that migraine is responsible for 4–5% of all YLDs throughout the world.

**Table 2:** Migraine in GBD2021 by World Bank income level (from [9])

World Bank income level	Disability-adjusted life years (DALYs)			Years lived with disability (YLDs)		
	Rank	Rate per 100,000	Proportion of all (%)	Rank	Rate per 100,000	Proportion of all (%)
All countries (from Table 1)	19th	550	1.49	4th	550	4.73
High	15th	616	1.83	8th	616	4.22
Upper-middle	14th	540	1.69	5th	540	4.65
Lower-middle	18th	564	1.43	4th	563	5.14
Low	28th	396	0.84	5th	396	4.13

In summary, GBD2021 says little that is new about headache in adults, but provides important new insights into headache in children. Where rankings have shifted slightly, these changes are reflections more of other diseases and of demographic shifts than of anything happening with headache. Whether measured in DALYs or YLDs, the ill-health burden of headache (608/100,000) has not changed since 2019, or indeed, since 2010 (603/100,000) [9]. Any hoped-for reduction through better care for headache [15] remains stubbornly unrealised.

The next major update of GBD is expected for 2023. In the meantime, how best to analyse headache data is the subject of intensive discussion and much alternative modelling. A substantial amount of new data is being entered from Global Campaign studies in Benin [16], Cameroon [17], India (New Delhi region) [18], Morocco [19] and Peru [20], which, along with previous studies, provide separate estimates for definite and probable migraine [12]. Given that, in studies that have reported both, they are approximately equal in prevalence [21], should they be considered equal in their contributions to burden – including YLD estimates? There are empirical data that will answer this question [22] for future GBD iterations.

Migraine in GBD has greatly distracted attention from tension-type headache (TTH) – more prevalent [8, 9] but undeniably less burdensome at individual level [23]. The so-called disability weights (DWs) attaching to migraine (0.441) and TTH (0.037) [24] reflect views expressed in a very large global survey that compared (ictal) health states attributed to the two (along with well over 200 health states attributed to other diseases), but described in lay terms and rather few words [24]. Are the DWs fair in their relative weighting of migraine and TTH? There are empirical data that will answer this question also [22].

## Data Availability

The data are publicly available on-line at https://vizhub.healthdata.org/gbd-compare/.

## Abbreviations

DALY: disability-adjusted life year
DW: disability weight
GBD: Global Burden of Disease (study)
TTH: tension-type headache
YLD: year lived with disability
YLL: year of life lost
